# Minute Rebond: A Simple Method for Making Lab-Scale Rebonded Foam and Its Application as a Novel Soilless Growing Media

**DOI:** 10.3390/polym17202770

**Published:** 2025-10-16

**Authors:** Michael S. Harris, Harry Charles Wright, Tom Lilly, Nathan Seithel, Chris Hayes, Julie Walker, Jacob Nickles, Duncan Drummond Cameron, Anthony John Ryan

**Affiliations:** 1 School of Mathematical and Physical Sciences and The Grantham Centre for Sustainable Futures, University of Sheffield, Sheffield S10 1TN, UK; harry.wright@sheffield.ac.uk (H.C.W.); a.ryan@sheffield.ac.uk (A.J.R.); 2 Department of Environmental and Earth Sciences, University of Manchester, Manchester M1 7DN, UK; 3 Vita Liquid Polymers, Wythenshawe M22 4SZ, UK; 4 Ball & Young, Corby NN17 4DU, UK; 5 Vitafoam, Manchester M24 2DB, UK; 6 School of Geography and Planning, University of Sheffield, Sheffield S10 1TN, UK; jacob.nickles@sheffield.ac.uk

**Keywords:** polyurethane foam, recycling, rebonded materials, soilless cultivation, circular economy, waste valorisation, sustainable materials, green engineering

## Abstract

Polyurethane foams (PUFs) utilised in the comfort industry generate substantial trim waste volumes requiring end-of-life management. Rebonding, one form of mechanical recycling, is a technique involving the mechanical breakdown and subsequent adhesion of PUF using polyurethane prepolymers yielding a recycled material. However, the limited investigation into the properties of rebond PUF constrains its potential for novel alternative uses, such as soilless plant-growing media. A laboratory-scale rebond production method has been developed, and a series of rebond PUFs produced to evaluate the influence of crumb size, density, prepolymer chemistry, and prepolymer loading on the properties of the rebond PUFs and their suitability as growing media. The results indicated that higher quality rebonds were obtained with larger crumb sizes (mixed or >7 mm), moderate amounts of prepolymer (4.5 to 7.5% by mass), and higher densities. Increasing density directly influenced plant growth-related properties, including reducing airflow, increasing water uptake through wicking, and increasing water retention through drainage alongside larger crumb sizes [>7 mm]. To demonstrate the method’s utility for rapid screening, a plant growth trial was conducted using density as the key variable. *Eruca sativa* plants grown in low-density rebonds exhibited comparable growth (leaf length, leaf width, and shoot fresh weight) to mineral wool, whereas medium- and high-density rebonds showed reduced growth. This study validates a lab-scale technique that enables the rapid optimisation of rebond PUFs for novel applications like soilless growing media.

## 1. Introduction

Global plastic production has reached an estimated 400 million tons in 2022 and is projected to maintain an exponential growth trajectory for the foreseeable future [1]. The inherent chemical resilience of most plastics results in slow natural degradation, necessitating effective end-of-life management to mitigate environmental accumulation [2]. With a growing emphasis on extended producer responsibility, where producers are incentivized to participate in the post-consumer collection and recycling of their materials once used [3], there is an increasing need for the industry to focus on how to manage their products at the end of life. While recycling has been a primary strategy for plastic waste management, it has primarily targeted thermoplastics, which are amenable to melt-processing and reformation into new materials [4]. Thermoset plastics, that form covalent bonds between polymer chains during their production, cannot be recycled in this way [5]. Therefore, recycling thermoset polymers presents a unique challenge for waste management.

Polyurethanes are an example of a polymer that can be produced as either thermoplastics or thermosets [6,7], with the majority produced being thermoset [8]. Polyurethanes can be synthesised with an expansive range of chemicals, allowing for materials with vastly different chemical and physical properties that can be utilised in a wide range of applications. For example, their polymer backbones can be a wide variety of polyols (e.g., poly(propylene oxide) glycol, castor oil, polycaprolactone, polycarbonate, etc.), isocyanates (aliphatic or aromatic), catalysts can include tin (e.g., Stannous Octoate or Dibutyltin dilaurate), or amine-based (e.g., pentamethyl diethylenetriamine, triethylenediamine, N,N,N′,N′,N″-pentamethyl diethylenetriamine, etc.) reagents, alongside a variety of fillers (e.g., CaCO_3_, graphite, clays, etc.), dyes, etc. [7]. When combined with further additional materials, such as surfactants and blowing agents, a wide range of polyurethane foams (PUFs) can be produced [6,7], which can be manufactured with diverse applications, from insulation to comfort [9]. However, the compositional complexity arising from the inclusion of diverse additives poses significant challenges for recycling these materials [10]. Flexible PUF, as a thermoset polymer, with low density and multiple additives, presents a unique waste management challenge, requiring substantial segregation for effective recycling or extensive landfill space for disposal [11,12]. With the demand for PUFs ever increasing [9], innovative recycling methodologies must be embraced to address these challenges.

**Figure 1:**
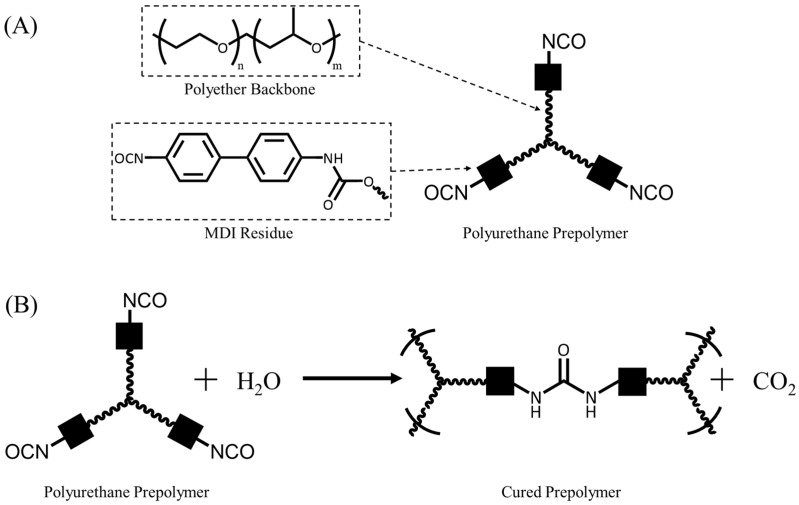
Diagram of the curing of polyurethane prepolymer with water. (**A**) The structure of a polyurethane prepolymer, where squares represent MDI residues, and the curvy lines represent the polyether backbone. (**B**) The curing of a polyurethane prepolymer with water to produce a cured prepolymer.

Rebonding, a form of mechanical recycling, represents a recycling approach for flexible PUFs [8]. This process involves grinding the solid waste into smaller particles, termed crumb, followed by size fractionation through sieving. The crumb is subsequently coated with a polyurethane prepolymer as an adhesive, compressed into an appropriate mould, and supersaturated steam (>140 °C) is passed through the compressed block. The steam cures the polyurethane prepolymer (Figure 1) and fixes the crumb in place, creating a rebonded PUF with a higher density than the constituent foam employed [7,12].

Rebonded PUF is currently utilised in various low-value applications, including sports mats [13], acoustic dampening [14], and underfloor padding [15]. The inherent heterogeneity of rebonded PUFs, resulting from the diverse composition of the crumb, leads to significant variability in the final product and hence its limited value. Identifying alternative applications, where the heterogeneous nature of these materials is not detrimental or may indeed be beneficial is key to widening their potential use and increasing the value of rebond PUFs and of rebonding as a route for recycling.

An area where inherent heterogeneity is already apparent is in soil structure. Soils are heterogeneous mixtures of inorganic and organic materials, in which their variation can be tuned for the growth of different species of plants [16]. Soils can be replaced by artificial materials, so long as they provide adequate properties for the species being grown in the material, such as airflow to allow respiration in the roots [17], water absorption to transport water to the roots [18], and structural stability to allow the plant to anchor to the substrate for growth [18]. Demand for such growing media is projected to exceed the capacity of existing materials, necessitating the development of novel alternatives [19]. This presents a potential application for rebond PUFs, which could act as an artificial growing medium for plants, widening the applications for this recycled material, especially in environments requiring a low-density media, such as green walls and roofing. PUF has already demonstrated its use in hydroponic applications as soilless growing media [20,21,22], and Benoit and Ceustermans have demonstrated rebonded PUFs from industry for the growth of herbs and use in gardens [23].

However, the hydrodynamic properties of rebonded PUFs are not well reported, limiting our understanding of its behaviour as a growing media, or other applications. In addition to the lack of characterisation of rebonded PUFs, there are no published methodologies for synthesising rebonded PUFs at laboratory scale, which complicates the standardisation of production for small-scale experiments.

Herein, we address the issues outlined above. Namely, the lack of a simple lab-scale (<500 g) process of producing rebonded polyurethane foam, the characterisation of rebonded PUF properties, and the evaluation of plant growth in the media.

This paper presents a simple methodology for producing lab-scale rebonded PUF, wherein the polyurethane crumb is sprayed with water, coated with a polyurethane prepolymer, compressed in a polypropylene container, and subjected to microwave heating for 60 s. The in situ production of steam from water within the crumb allows complete curing of the rebond block, regardless of the density.

To validate the method, a series of laboratory-scale rebonded PUFs were produced via the microwaving technique to characterise their hydrodynamic and airflow properties. Finally, to demonstrate the key advantage of this technique, the ability to rapidly prototype and screen material properties for specific applications, a plant growth trial was conducted. This trial serves as a proof-of-concept, using density as a variable to quickly assess the suitability of these materials as a soilless growing medium and to showcase the method’s effectiveness.

## 2. Materials and Methods

### 2.1. Materials

All foam crumbs and prepolymers were supplied by Vitafoam (Middleton, UK), with specific formulations withheld. Foam crumb was formed from trim scrap from comfort foam production with a range of initial foam densities typical of European bedding. Industrial recipe polyurethane prepolymers were formulated from Methylene diphenyl diisocyanate (MDI) and a polyether polyol containing either 15% or 73% ethylene oxide (EO) content, and blends of the two were provided for other required compositions. The 15% EO prepolymer formulation is typically used for commercial rebonding. For lab-scale rebonding, a Décor Microwave Rice Cooker 2.75 L (The Decor Corporation Pty. Ltd., Melbourne, Australia) was used as a container, a polypropylene disk for compression, and a 20 L white freestanding digital 800 W microwave oven for microwave heating.

Rucola (*Eruca sativa*) seeds were purchased from Natures Root (Enfield, UK). In addition, 5% sodium hypochlorite (NaClO) was purchased from Reagecon (Shannon, Ireland), and 99.8% ethanol was purchased from Fisher (Loughborough, UK). Nutrient solutions were purchased from VitaLink (Coventry, UK). Mineral wool was purchased from Grodan (Roermond, The Netherlands).

### 2.2. Rebonding Method

**Figure 2:**
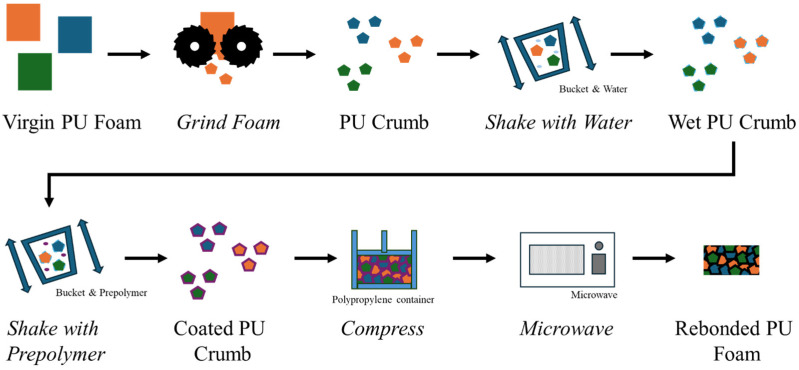
Diagram of the method for producing rebonded foam via a microwave at lab scale.

PUF crumb was obtained from industrial sources but can be generated locally through the comminution (grinding/crushing/cutting) of virgin PUFs. The laboratory-scale rebonding process is shown in Figure 2 and comprises the following steps:

1. Wetting PUF Crumb

To facilitate prepolymer curing via steam generation, a controlled quantity of deionised water was introduced into the PUF crumb. The desired mass of PUF crumb was transferred to a polypropylene mixing container. Deionised water was applied via a spray bottle (for this study, 1 to 3 times the stoichiometrically amount of water was used). The container was sealed, and the mixture was manually agitated (shaken) for 30 s to ensure uniform water distribution throughout the PUF crumb, and the container lid was removed.

2. Prepolymer application

A predetermined mass of polyurethane prepolymer was then dispensed via pipette onto the wet PUF crumb in the container. This work utilised 4.5–7.5% by weight of dry crumb, although consideration should be given to the PUF crumb size utilised, as a smaller crumb with a higher surface area will require greater amounts of prepolymer. Once added, the lid of the container is closed, and the foam is shaken by hand for 30 s to ensure a uniform distribution of the prepolymer. Note the prepolymer will begin curing in the presence of the wet crumb, meaning it cannot be stored for later use. Any crumb coated in prepolymer must be immediately used.

3. Compressing PUF Crumb

The prepolymer-coated PUF crumb was transferred to a microwave safe container and compressed with a lid to a desired height/density. The height or density is determined by the mass of crumb added to the microwave safe container of fixed volume; in this work, 40, 80, or 120 g of crumb was compressed into a 16 × 5 cm cylindrical container. Once compressed, ensure the perforated lid is effectively closed and will not re-open during the microwaving process. Layered compression (i.e., sequential addition and compression of crumb) should be avoided to prevent the formation of density gradients within the rebonded foam.

4. Microwaving PUF Crumb

The container, containing a compressed coated crumb, is then placed into a standard domestic microwave for heating. Microwave irradiation was conducted at a power output of 800 W for a duration of 60 s, allowing for full steaming of the PUF crumb without overheating. The microwaving duration will vary depending on the power of the microwave used. Following irradiation, the rebonded PUF was allowed to cool for 30 s prior to removal from the container. Successful rebonding yielded a cohesive piece of PUF that could be manually extracted from the container in a single piece.

### 2.3. Rebonding Material and Equipment Considerations

To produce a low-grade rebonded PUF product, diverse PUF crumb sources, residual materials such as foam skin (the outer edge of a produced foam), polypropylene covers (typically used in the continuous production of PUFs to prevent sticking to conveyor belts), baler straps (polypropylene wire used to hold mass quantities of foam together for transport), etc., can be utilised. However, this approach introduces batch-to-batch variation and produces low-quality rebonded PUFs. For a higher quality product, greater attention should be paid to the foam crumb utilised. Homogeneous materials are best achieved using single-source, high-quality crumb, devoid of extraneous materials. However, the use of these materials will likely incur increased material acquisition and processing costs, which should be considered when synthesising these rebonds.

Manual agitation in polypropylene buckets with lids was employed for mixing crumb and prepolymer constituents. For larger-scale production, automated mixing systems could be implemented; however, this is not required at laboratory scale. Regular cleaning or the use of disposable mixing equipment is recommended to mitigate prepolymer buildup.

It should be ensured that all equipment and materials utilised in the microwave irradiation process are microwave safe for safety. This work uses microwave-safe polypropylene materials throughout. A steam escape to prevent any pressure build up within the system should be employed. Additionally, the use of polypropylene for the steamer ensures the effective release of the rebond once cured.

The final considerations for this work are for safety. PUF is not microwave transparent and will absorb microwave energy, potentially leading to scorching or combustion. This can be mitigated by using flame-retardant foams; however, this does not stop scorching and simply slows burning. Additional water can be used to mitigate these issues but again does not guarantee preventing scorching or burning. Users should determine the minimum amount of time required to sufficiently cure a rebond without scorching or burning based on their specific reaction system (microwave/crumb/prepolymer).

In domestic microwaves, the rotating turntable must be inspected before each rebond synthesis to ensure the effective rotation of the material, and that the container does not catch on the sides of the microwave, preventing it from spinning. In either case, if the foam cannot effectively rotate, there is a higher likelihood of scorching and burning, even with the controls described above.

### 2.4. Rebond Method Validation via Experimental Design

To reduce the experimental complexity of the investigation, we utilised a design of experiment (DoE), a structured and systematic framework to experimental design enabling an efficient exploration of formulation space and component interactions through the selection of experimental runs. For formulating polyurethane foams, DoE has demonstrated its use as a powerful tool to optimise parameters and gain an idealised set of results with a minimum number of experiments [24,25,26]. In this study, DoE was employed to investigate the influence of key process variables on the quality and hydrodynamic properties of microwave-cured rebonded PUFs intended for hydroponic applications.

The properties of rebonded PUFs are influenced by several factors, including crumb size, prepolymer composition, prepolymer amount, and compression, that affect the final density. An understanding of how these factors influence the final product is essential for optimising the rebonded foam. For example, crumb size affects the surface area available for prepolymer binding, influencing the mechanical integrity and porosity of the rebonded foam. Prepolymer composition, specifically the ethylene oxide (EO) content, modulates the hydrophilicity of the adhesive, impacting water retention and nutrient uptake. The amount of prepolymer used determines the degree of crumb binding, with insufficient prepolymer leading to weak rebonds and excess prepolymer resulting in hard beads within the product. Finally, compression or density affect the pore structure and airflow properties of the rebonded foam, influencing water drainage and aeration.

#### Design of Experiment Problem Statement

1. Utilisation of microwave irradiation to produce laboratory-scale rebond foam is a novel technique with multiple possible factors that could contribute to the resultant quality of the rebond foam, subsequently affecting the hydrodynamic properties of the foams.
2. Selection of response variable, factors, and factor ranges.

Four independent factors were chosen to answer the problem statement. Specifically, those that could be controlled in industrial application, and therefore would be relevant for application. Crumb mass (40, 80, or 120 g) was chosen as the first variable, as within this experiment, a fixed compression height was utilised; therefore, altering the mass of the crumb used would result in varying rebonding densities. Following this, the prepolymer mass (5.5 to 7.5% mass by dry weight of foam) and EO content (15 to 73% of the polyol) were also chosen. Finally, crumb size (Crumb used as provided; “Full Mixed”, Crumb filtered to <7 mm, and crumb filtered to >7 mm) was also chosen as a factor. The responses selected for analysis were chosen based on their relevance to the application of rebonded PUFs as growing media. The responses selected were the following: density, airflow, water retention capacity after wicking, and water retention capacity after draining. Specific formulations for rebonds can be found in the Supplementary Table S1.

### 2.5. Rebond Foam Physical Property Characterisation

Polyurethane foam crumb was separated by size via riddling, where the mixed sized (Full Mixed) crumb was placed onto a mesh (7 mm square holes) and shaken until crumb < 7 mm had passed through into a container below the mesh, and crumb > 7 mm remained.

Rebonds from the microwave technique were produced at a height of 5 cm and diameter of 16 cm. These foams were cut using a foam saw to produce two 50 mm × 50 mm × 100 mm samples used for analysis. To dry the foams, they were placed in a 70 °C oven for 16 h and allowed to cool before further use.

Density was measured from the mass of a dry rebond, and dimensions were measured using calipers to provide the density. All values are quoted in kg·m^−3^. The mean as well as standard error is reported.

Airflow was measured on a custom air flow apparatus in accordance with ISO 7231:2010 [27] using dry rebond cut to 25 mm × 50 mm × 50 mm.

The water absorption characteristics of rebond foams were assessed using a bespoke set up based on Schulker et al. [18] for the analysis. Specifically, dry rebonds were immersed into a constant height (25 mm) of water maintained by a pump and reservoir and allowed to wick for 180 min until equilibrium. The mass of the rebond was measured at multiple time intervals, with the equilibrium mass after 180 min reported. To normalise the data, the mass of the water was divided by the cross-sectional area of the rebond (50 mm × 50 mm), yielding a measure of water absorption expressed in g_H2O_·dm^−2^.

The water retention capacity of the rebonded polyurethane foams, following drainage, was determined using the following procedure. Dry rebonded PUF samples were fully submerged in deionized water for 16 h to ensure complete saturation. The samples were then removed and allowed to drain vertically under gravity for 24 h to reach drainage equilibrium. The mass of the rebond was taken periodically over time, with the mass after 24 h of drainage reported here. This mass of the water in the rebond is then divided by the volume (50 mm × 50 mm × 100 mm) of the rebond to produce a value of g_H2O_·dm^−3^.

For Scanning Electron Microscopy (SEM), a 10 mm × 10 mm × 2 mm sample is cut from dry virgin foam using a scalpel and sputter coated with 10 nm gold/platinum using a Quorum Q150TES Au/Pd coater (Quorum, Laughton, England). Images were produced using an Electron Microscope (SEM) TESCAN MIRA3 SC + OI EDS (TESCAN group, Brno, Czech Republic) in the secondary electron mode, at 10 kV, wide field, a working distance of 10.00 mm, and a magnification of 197×.

Foams’ effective open cell fraction was determined via a method described by Yasunaga et al. [28]. Namely, the number of open windows (N_open_), closed windows (N_closed_), partially open windows (N_part_), and windows containing pinholes (N_pin_) was counted from an SEM image. Once tallied, the effective open cell content (p_eff_) was determined by Equation (1), as follows:

(1) $$p_{eff}=\frac{N_{open}+0.5\times N_{part}}{N_{open}+N_{part}+N_{pin}+N_{closed}}$$

### 2.6. Plant Growth Trial

For planting, Rucola (*Eruca sativa*) seeds were sterilised in a laminar flow hood under aseptic conditions. Sterilisation involved the seeds being vortexed for 30 s with 25 mL of 99.8% ethanol. The seeds were then surface sterilised with 1% NaClO solution and vortexed for one minute. One minute with 30 mL deionised water to remove residual NaClO solution.

Seeds were planted in rebonded foam blocks (100 mm × 100 mm × 50 mm), with one seed planted in each corner (at least 10 mm from the edge), and one in the centre (5 seeds per block). All seeds were planted at least 1 cm deep with the aid of a scalpel to cut an entrance into the foam. Five replicate blocks of foams were seeded at each density for a total of 25 plants per foam density. Each replicate block was placed in a square petri dish (100 mm × 100 mm× 10 mm) to prevent run-off during watering.

Mineral wool was used as a control, with 9 mineral wool starter plugs (25 mm × 25 mm × 40 mm) placed in a square petri dish (100 mm × 100 mm× 10 mm), and one seed was placed in each plug. Five replicates of mineral wool were used for a total of 45 plants.

Plants were grown in the media for 3 weeks in a growth chamber (Grobotics Instruments Grobot α) at a constant temperature of 22 °C, 14 h light, and 10 h dark. The media were watered at the base of the media to 80% saturation daily with nutrient solution (Vitalink Max Bloom A + B Solution, made up to a concentration of 2 mL·L^−1^ of each A and B solutions in deionised water), with a pH of 5.8 and electrical conductivity of 0.93 dS·m^−1^. Following the three-week growth period, plant morphology and physiology were assessed.

Germination was determined from a yes/no binary if least cotyledons had emerged from the media. Leaf length and width were recorded for all leaves exceeding 1 cm. The largest new true leaf was used for an operational quantum yield measurement (measurement of the efficiency of photosystem II, F_v_/F_m_, in light conditions), using a FluorPen FP 100 (PSI (Photon Systems Instruments), Drásov, Czech Republic). Fresh shoot mass was determined for each plant.

For all subsequent statistical analysis, the block (i.e., the foam block or the tray of mineral wool plugs) was treated as the independent experimental unit. To achieve this, measurements from all individual plants within a single replicate block were first averaged to generate a single mean value for that block. These replicate means (5 replicates for each treatment) were then used to calculate the overall mean and standard error for each foam density and the mineral wool control. This approach accounts for variability in germination and ensures that each replicate contributed a single data point to the final analysis.

### 2.7. Analysis and Statistical Techniques

The determination of the DoE formulation points was conducted using JMP^®^ Pro 17.0.0 (SAS Institute Inc., Cary, NC, USA). Modelling physical property responses was also conducted in JMP^®^ Pro 17.0.0 using stepwise linear regression and k-fold cross validation (k = 5) with a hereditary restriction. All residuals were checked for normality and homogeneity of variance.

Stepwise linear regression is a modelling method that systematically explores model variations through the stepwise inclusion or exclusion of individual variables, based on their statistical significance [29]. In this work, a second-order response model was explored, observing individual and cross-products of all variables. K-fold cross-validation is a technique for enhancing model robustness by randomly splitting the data into k subsets, using each subset once as a validation set while the remaining subsets are used to train the model [30]. For this work, the model providing the best R^2^ value to validate the model was chosen.

Statistical analysis was performed using JMP^®^ Pro 17.0.0. Data distribution was first assessed with histogram plots and the Anderson-Darling test to determine normality. Normally distributed data were analysed using ANOVA, with significance levels determined by the Tukey test. For non-normally distributed data, the Kruskal–Wallis test was applied.

Graphics were generated using RStudio 2024.12.1+563 (Posit, Boston, MA, USA) with R package 4.4.3, utilising the ‘tidyverse’, gridExtra’, and ‘ggpmisc’ libraries. Linear fits were performed using the geom_smooth function (method = ‘lm’). Polynomial fits were conducted with method = ‘lm’ and formula = y ~ poly(x, 2). Scatter plots were created using the geom_point, bar charts were created using geom_bar, and box plots were generated using the geom_boxplot.

## 3. Results and Discussion

### 3.1. Effect of Parameters on Quality of Rebonds

The microwave fabrication process yielded solid, well-bonded, PUF disks that were easy to remove from the reaction vessel, demonstrating effective inter-crumb binding. A single foam specimen was discarded due to inadequate binding, attributed to suboptimal mixing during the prepolymer application stage.

Foams that contained higher prepolymer contents (7.5%) had a higher likelihood to form hard prepolymer beads within the rebond. This phenomenon is likely attributable to the high viscosity of the prepolymer preventing efficient mixing, resulting in the formation of prepolymer droplets within the rebond. Once steamed, these turn into hard beads. The ethylene oxide (EO) content of the prepolymer and the crumb size did not affect the overall quality of the rebonded foams or the incidence of bead formation.

**Figure 3:**
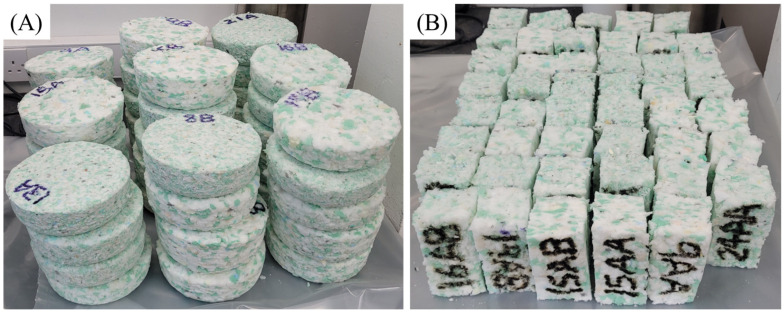
(**A**) Produced rebond foams from DoE. (**B**) Cut samples for testing.

Rebonds then were sectioned using a foam saw to allow for smooth uniform cuts to be made (Figure 3), producing two test specimens with dimensions of 50 mm × 50 mm × 100 mm Rebond foams made with lower prepolymer content (4.5%), lower crumb mass (40 g), and smaller crumb sizes (<7 mm) were susceptible to tearing during sectioning, which could be prevented by slowly cutting through the rebond.

### 3.2. Effect on Crumb Mass on Final Density

**Figure 4:**
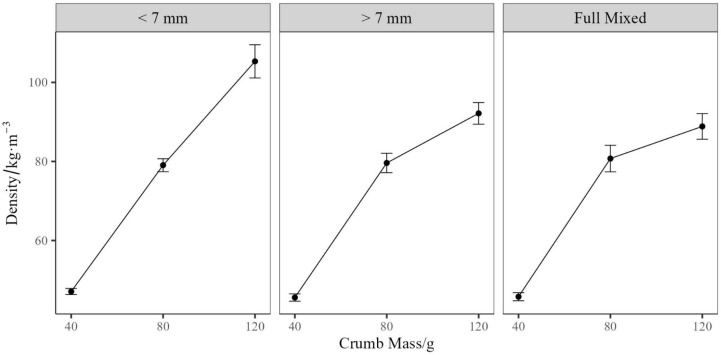
Plot of crumb mass vs. density, split by the crumb sizes < 7 mm, >7 mm, and Full Mixed. The value is the mean of four replicates, with error bars representing standard error.

Figure 4 displays the relationship between crumb sizes, crumb mass, and the rebonded foam. Density did not scale linearly with crumb mass for foams containing mixed crumb sizes, or >7 mm crumbs. In contrast, a linear relationship was observed for foams containing a <7 mm crumb. This deviation from the expected linear relationship is attributed to the inefficient packing of the PUF crumb, with larger pieces packing less efficiently, resulting in intra-crumb voids on compression. Following microwave treatment and demolding, the crumb expands, increasing the volume of the rebond and causing a corresponding reduction in the final density. This results in diminishing returns in the higher densities when larger crumb sizes are utilised, even in mixed crumb systems. This can be alleviated using smaller crumbs, which allows for the more efficient packing of smaller particulates. However, there is likely an upper limit of the density that can be achieved using smaller particulates, approaching that of the strut density of the PUF. Therefore, consideration should be given to the desired final density of rebonded foams, with higher densities requiring smaller crumbs, compared to lower densities. Consideration could also be given to the initial density of the crumb being utilised, alongside the amount of prepolymer, as with higher density foam and more prepolymer, higher density rebonds can be produced.

### 3.3. Modelling Airflow

**Figure 5:**
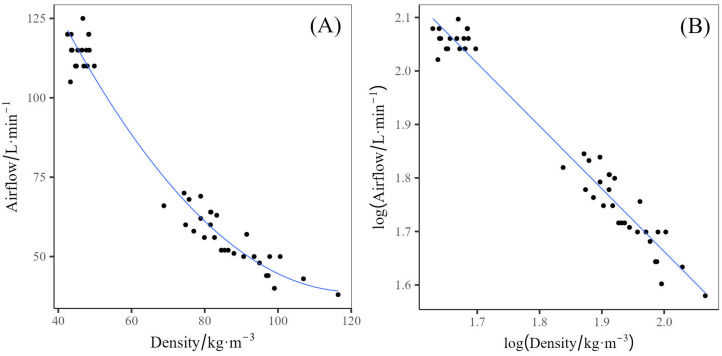
(**A**) Airflow as a function of Density, with the solid line indicating a polynomial (n = 2) fit of the data, (**B**) log(Airflow) as a function of log(Density) with the solid line indicating a linear fit.

The airflow through the rebond block exhibited an inverse correlation with the density of the rebond produced (Figure 5), regardless of crumb size, prepolymer content, or EO content. Rebonds themselves have high airflow rates, with the low density (crumb mass of 40 g) achieving 105–125 L·min^−1^ of airflow, and the high density (120 g) achieving 38–64 L·min^−1^. The airflow through virgin polyurethane foams is also inversely related to the density and closed cell content of the foam, with higher densities and more closed cells resulting in lower airflows [24]. The comparatively high airflow rates observed in the rebond foams, even at higher densities than typical virgin polyurethanes, were attributed to the discontinuous nature of the rebonds, allowing for the gaps between the crumb to allow higher airflows. This effect is reduced with the higher density rebonds as the packing of the crumb is forced closer together, reducing the effective gaps in the rebond.

**Figure 6:**
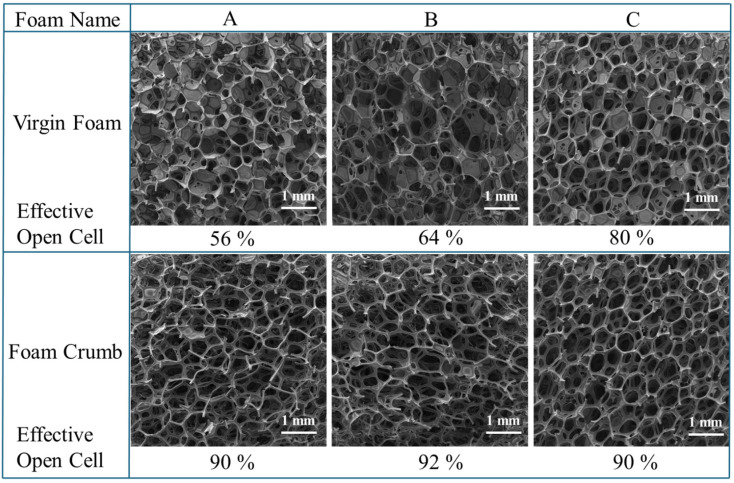
Effective open cell fraction change from before and after crumbing foam for foams labelled A, B, and C.

Additionally, the comminution process employed to produce foam crumb significantly increases the number of open cells in a foam. Figure 6 shows three different foams, both in their virgin form as well as post comminution, as well as their open cell content. The processed foams had over 90% effective open cells, enabling improved airflow through the structure and resulting in higher airflow rates compared to the virgin foam used for rebonding.

### 3.4. Modelling Water Wicking Mass

**Figure 7:**
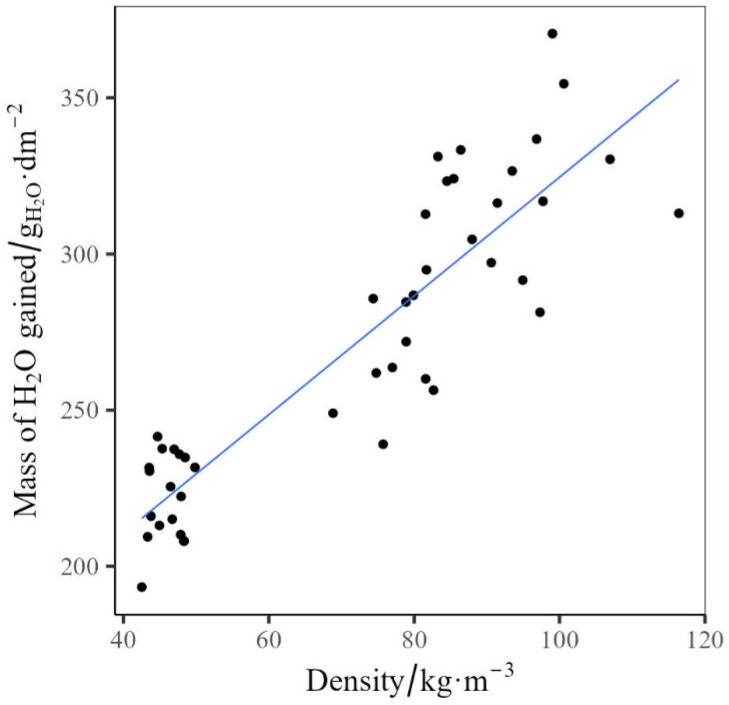
Mass of water gained as a function of Density with the solid line indicating a linear fit.

The maximum water uptake was used to assess the influence of crumb size, crumb mass, prepolymer mass, and EO content on the wicking capacity of the rebond foams. Of the factors analysed, the density of the rebond foam had the highest influence on the wicking capacity. This was found to be a proportional trend between the factor and response (Figure 7).

This is likely attributable to the compression of the foam cells, resulting in a more effective capillary pathway for water transport into the rebond. Additionally, the increased mass of material provides a greater number of cells for the water to be wicked into.

The crumb size, prepolymer mass, and EO content did not contribute significantly to the models produced. It is acknowledged that this relationship is likely more complex than only being influenced by density, as the hydrophilicity of the foam crumb will impact the final material. In this work, an industrial sample of crumb was taken from a heterogeneous mixture of polyurethane foams. This compositional complexity introduces variability in the water wicking behaviour. Therefore, this study provides a realistic representation of industrial rebonded foam products and highlights the potential for future research to investigate the effects of bespoke polyurethane crumb mixtures on water wicking capacity.

### 3.5. Modelling Water Draining Mass

**Figure 8:**
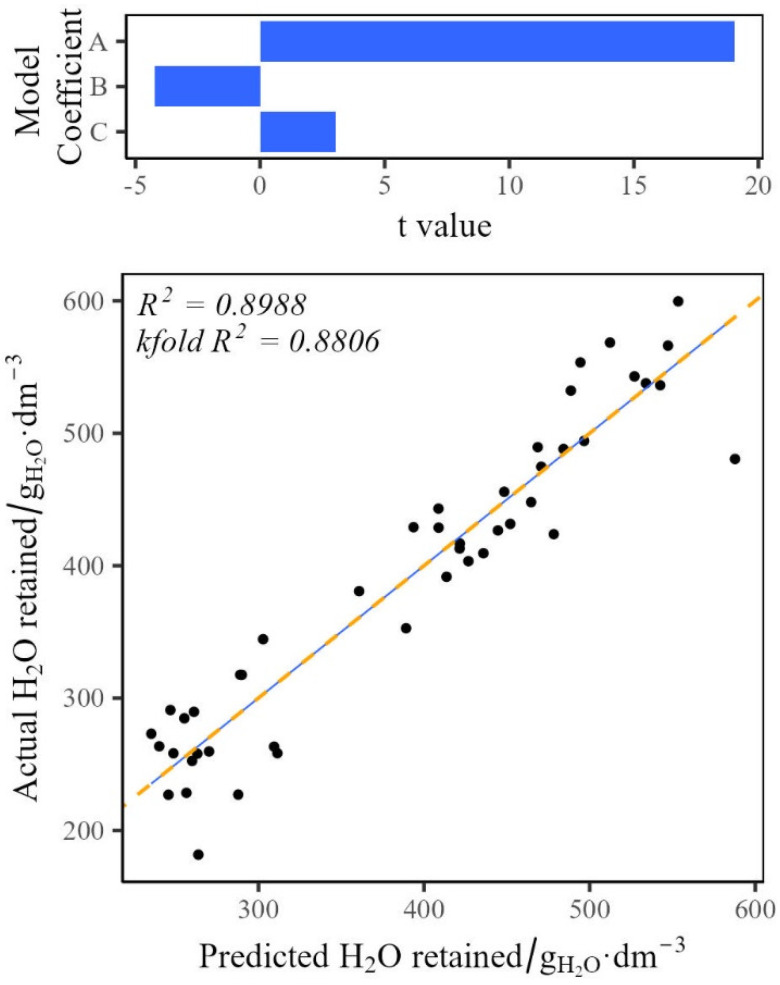
Model fitting for water mass retention after 24 h. The effects of the model coefficient shown above, where A is Density, B is Crumb Size, and C is the Intercept. The prediction graph below shows predicted and actual values, with the solid blue line indicating a linear fit, and the dashed orange line showing a y = x linear fit.

The drainage of water from the rebonded foams exhibited a more complex behaviour than the density or the wicking characteristics (Figure 8). Stepwise regression was used, and a second order model was found to best fit these data (Equation (2)). The model uses a fitting coefficient for each response (density and crumb size) as well as a random error parameter accounting for the remaining errors in the model.

Equation (2) demonstrates that the water retained within the rebond system is based upon a combination of the crumb size and density. The model indicates that the use of the smaller crumb (<7 mm) or mixed crumb has a negative effect on the water mass retained, whilst the large (>7 mm) crumb has a positive effect. Density has a positive correlation regardless of crumb size.

$$y=\beta_{C}\times Crumb\;Size+\beta_{D}\times Density+\varepsilon$$

(2) $$Crumb\;Size=\left\{\begin{array}{c} <7\;mm=-1\\ Full\;Mixed=-1\\ 7\;\mathrm{mm}=1 \end{array}\right.$$

The observed increase in water retention after draining as a function of increased density and crumb size is likely attributed to the increased number of “water reservoirs” occurring in these foam structures. The increased density provides more polymer and a higher surface area for water to interact and adsorb within the rebond. Larger crumb sizes likely result in fewer contact points between individual pieces of crumb, thereby reducing the number of pathways available for water to easily flow out of the foam.

### 3.6. Growth of Plants in Rebonds

**Figure 9:**
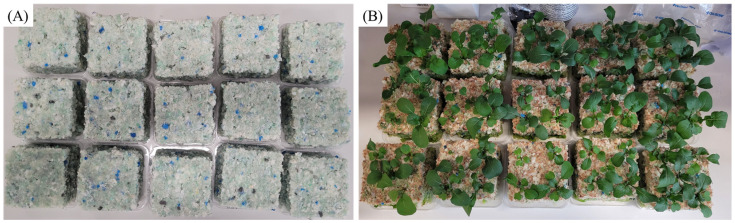
(**A**) Image of different density rebonded PUFs used for Rucola growth. (**B**) Rucola grown in rebonded PUFs after 3 weeks. In both images, the low-density rebond is on the top row, medium density rebond in the middle row, and high density rebond in the bottom row.

To demonstrate the utility of the lab-scale method for rapidly screening material properties for a target application, a growth trial was conducted. Density was chosen as the primary variable for this proof-of-concept, as the preceding data established it has the most significant influence on both airflow and hydrodynamic properties critical for a growing medium. Rebonds were produced using a mixed crumb size, low EO content polyol (15%), and 6% prepolymer mass. These factors remained constant at each density. Rebonds were produced with either 40 g, 80 g, or 120 g of full mixed foam crumb, resulting in rebonds labelled as Low Density (40 g, LD), Medium Density (80 g, MD), or High Density (120 g, HD). These samples were used for the growth trial, as described in Section 2.6 of methods, and the resultant plants are pictured in Figure 9. Rucola was selected as the species of interest for this trial due to its rapid germination, short growth cycle, and growth expertise within the research group. An additional growth in mineral wool (MW) was also undertaken to create a comparison to a material already utilised as a plant growth media.

**Figure 10:**
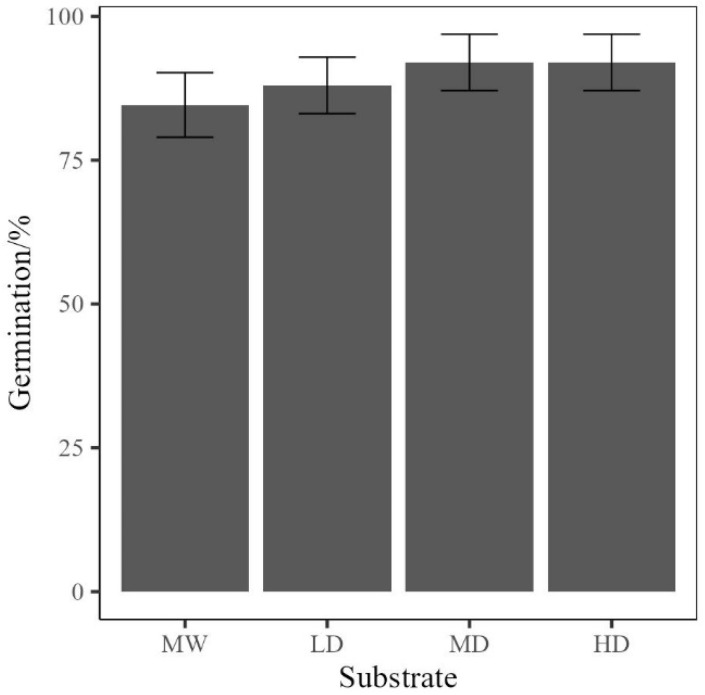
Plot of germination vs. media. The value is the mean of five replicates, with error bars representing standard error. MW is Mineral wool, LD is low-density rebonded PUF, MD is medium-density rebonded PUF, and HD is high-density rebonded PUF.

Germination was measured as the first indicator of rebonding as a viable growth media, with rucola achieving an average of at least 80% germination in every media utilised for growth (Figure 10), with the medium- and high-density rebonds having an average of over 90%. This primarily indicates that the use of rebonds is viable for the germination of plants, where multiple densities can be utilised without a reduction in germination rates below mineral wool (ANOVA; F [3,4] = 1.8127, *p* = 0.612).

After three weeks of growth, rucola plants were measured for their largest leaf width, length, and quantum yield as indicators of plant health and yield. Finally, the total shoot fresh mass of each plant was also measured.

**Figure 11:**
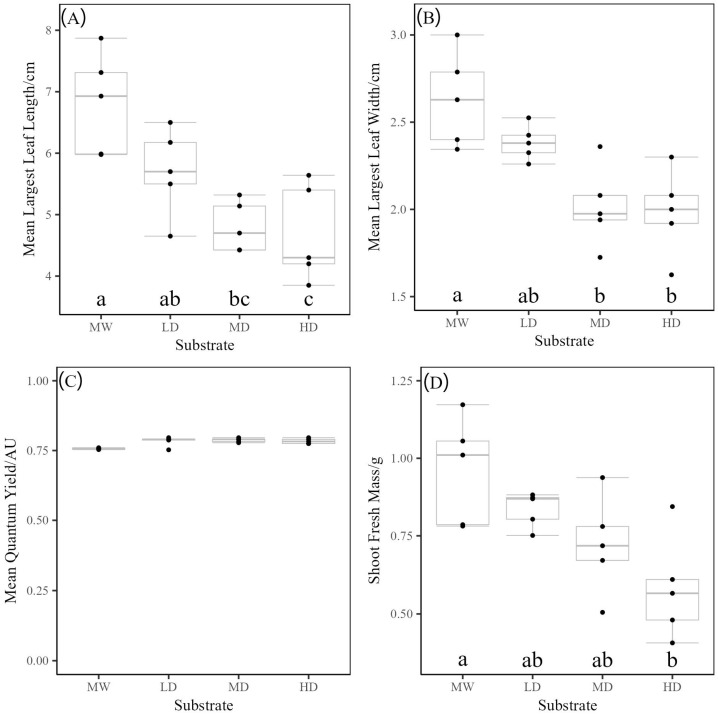
The effect of density on Rucola plants grown in media. (**A**) The effect of media on Rucola mean leaf length. (**B**) The effect of media on Rucola mean leaf width. (**C**) The effect of media on rucola mean quantum yield. (**D**) The effect of media on Rucola shoot fresh mass. Media are denoted as HD (High density), MD (Medium Density), and LD (Low Density), with mineral wool (MW) included as a comparative control group. The box is created from the first to the third quartile, and the horizontal line through the box indicates the median. Whiskers represent the maximum and minimum values within 1.5 times the interquartile range. Letters indicate significance via Tukey test, where data are normally distributed.

For the largest leaf width, length, and wet mass, the use of mineral wool as a growing media outperformed all MD and HD rebonded PUF media, with low-density rebonded PUFs achieving comparable growth (Figure 11, Table 1). When analysing Tukey’s HSD test on each parameter, low-density rebonded foam performs similarly to mineral wool, showing no significant difference in all measurements. In contrast, high-density foam exhibited significantly smaller leaf sizes and a lower overall wet mass when compared to mineral wool, and its leaf length was also shorter than that of low-density foam. Medium-density rebond foam is comparable to both low- and high-density foam, and only comparable to mineral wool in wet mass. Overall, this demonstrated a trend that with higher density foams, a reduction in plant size is seen.

A final measurement, quantum yield, was made as it has been used as an indicator of plant health [31]. Mineral wool demonstrated an average of 0.76, while high- and low-density rebonded foams had values of 0.78, and medium-density rebonded foam showed a value of 0.79. This slight increase in quantum yield may be attributed to the possibly enhanced water-holding capacity of the foams, which could help mitigate the risk of drought stress in the plants [32]. However, the wide range of quantum yield values observed for the low-density rebonded foam suggests that the data should be primarily interpreted that, overall, the health of plants grown in all media remains generally good.

Overall, the growth of Rucola in rebonded foams demonstrates that they are viable growth media, with potential for further optimisation for future plant growth.

## 4. Conclusions

This study presents a laboratory-scale method to produce rebonded polyurethane. Rebonded foams were successfully produced by microwave-assisted curing of damp polyurethane foam crumbs coated with a polyurethane prepolymer under compression. This method facilitates the rapid and cost-effective production of small-scale rebonded foam specimens, enabling the systematic investigation of multiple factors influencing rebonding properties. 

To demonstrate a use case, this method was used in the design of an experiment, looking at the foam crumb size (Full Mixed sizes, <7 mm, >7 mm), crumb mass (40, 80, or 120 g), prepolymer ethylene oxide content (15 to 73%), and prepolymer mass (4.5 to 7.5%), and their effects on the physical properties of rebonded foam produced. These rebonds were evaluated for their quality, airflow, and hydrodynamic properties (wicking and draining) to assess their potential for use as growing media.

Rebonding quality was evaluated based on the ease of demoulding, processability, and the presence of hard prepolymer beads. The quality of the rebonds was reduced by a combination of lower prepolymer mass, crumb size, and crumb mass, resulting in an inferior distribution of prepolymer over a larger surface area of the crumb, and the poorer binding of the final product. This resulted in the increased likelihood of the tearing of the rebond when trying to cut it to shape. Conversely, increased prepolymer masses resulted in a higher likelihood of hard beads forming in the rebonding due to inefficient prepolymer distribution.

The density of the rebonded foam was influenced by the crumb size and mass used. With increasing crumb mass, higher rebonding densities could be produced. However, due to the relaxation of the crumb after the curing of the prepolymer, those made with larger crumb sizes resulted in lower densities than would have been expected. Therefore, to be able to access high rebonding densities, smaller crumb sizes should be utilised.

Density was the most influential factor on airflow, the wicking of water into rebonds, and the draining of water out of rebonds. Crumb size also influenced the draining, and therefore a model was produced to account for this. These effects are attributed to the influence of density and crumb size on the formation of capillary pathways for water transport and retention.

Rucola (*Eruca sativa*) plants were then grown in rebonded foam at three different densities and compared to a mineral wool control. Rucola germinated over 80% in all media, with the low-density foam exhibiting growth performance that matched mineral wool. The higher density foams showed suppressed growth when compared to mineral wool and LD foam, which may be due to the amount of water contained and airflow in the different densities of the rebond.

In conclusion, this study successfully establishes a novel, microwave-assisted technique for producing rebonded polyurethane foam at a laboratory scale. The key advantage of this method is its capacity for rapid prototyping and the screening of material properties. We demonstrated this by systematically showing that density was the most influential factor on the foam’s hydrodynamic and airflow characteristics. The subsequent plant growth trial served as a successful proof-of-concept, confirming that the method can be used to quickly identify promising formulations for specific applications, such as soilless growing media. The finding that low-density rebonds perform comparably to mineral wool not only highlights the potential of this recycling pathway but also validates the efficacy of our screening approach. This rapid, adaptable, lab-scale process opens the door for the efficient optimisation and exploration of rebonded PUFs for a variety of novel, higher-value applications.

## Figures and Tables

**Table 1:** Statistical analysis of rebond density effects on Rucola germination, mean leaf length, mean leaf width, mean quantum yield, and wet mass. Significance was analysed via ANOVA except for germination and mean quantum yield, which were analysed via the Kruskal–Wallis test. A total of 120 seeds were planted.

Measured Value	DF (Between, Within)	Sum of Squares	Chi Squared	R^2^	F	*p*
Germination/%	3, 16	-	1.8127	-	-	0.6122
Mean Largest Leaf Length/cm	3, 16	36.56	-	12.16	10.91	<0.0001
Mean Largest Leaf Width/cm	3, 16	3.03	-	1.01	5.20	0.0022
Mean Quantum Yield/AU	3, 16	-	47.21	-	-	<0.0001
Shoot Fresh Mass/g	3, 16	2.06	-	0.69	4.21	0.0075

## Data Availability

All primary and supplementary data used in this study is available under a CC BY NC 4.0 license and is available at https://doi.org/10.15131/shef.data.30285556 (accessed on 2 October 2025).

## Supplementary Materials

The following supporting information can be downloaded at: https://www.mdpi.com/article/10.3390/polym17202770/s1, Table S1: Rebond polyurethane foam formulations for experiments.
