# Supplementary material for: Minute Rebond: A Simple Method for Making Lab-Scale Rebonded Foam and Its Application as a Novel Soilless Growing Media
Source: Polymers (Basel). 2025 Oct 16;17(20):2770. doi: 10.3390/polym17202770 (PMC12566798; doi:10.3390/polym17202770)
Supplement: Supplementary file 1 [file polymers-17-02770-s001.zip › polymers-3884726-supplementary.pdf]

Table S1: Rebond polyurethane foam formulations for experiments

Batch Name	EO Content / %	Prepolymer Mass / %	PU Crumb Mass / g	Crumb Size
1AA	73	7.5	40	< 7 mm
1AB	73	7.5	40	< 7 mm
2AA	73	4.5	80	Full Mixed
2AB	73	4.5	80	Full Mixed
3AA	73	7.5	120	Full Mixed
3AB	73	7.5	120	Full Mixed
4AA	15	7.5	40	> 7 mm
4AB	15	7.5	40	> 7 mm
5AA	15	4.5	80	> 7 mm
5AB	15	4.5	80	> 7 mm
6AA	73	7.5	80	> 7 mm
6AB	73	7.5	80	> 7 mm
7AA	73	4.5	40	< 7 mm
7AB	73	4.5	40	< 7 mm
8AA	15	4.5	120	< 7 mm
8AB	15	4.5	120	< 7 mm
9AA	44	7.5	120	> 7 mm
9AB	44	7.5	120	> 7 mm
10AA	15	4.5	40	Full Mixed
10AB	15	4.5	40	Full Mixed
11AA	73	4.5	120	> 7 mm
11AB	73	4.5	120	> 7 mm
12AA	15	7.5	80	Full Mixed
12AB	15	7.5	80	Full Mixed
13AA	15	7.5	120	< 7 mm
13AB	15	7.5	120	< 7 mm
14AA	34.43	4.5	120	Full Mixed
14AB	34.43	4.5	120	Full Mixed
15AA	15	6	120	> 7 mm
15AB	15	6	120	> 7 mm
16AA	44	7.5	40	Full Mixed
16AB	44	7.5	40	Full Mixed
17AA	15	6	40	< 7 mm
17AB	15	6	40	< 7 mm
18AA	73	6	40	Full Mixed
18AB	73	6	40	Full Mixed
19AA	44	4.5	40	> 7 mm
19AB	44	4.5	40	> 7 mm
20AA	44	6	80	< 7 mm
20AB	44	6	80	< 7 mm
21AA	73	6	120	< 7 mm
21AB	73	6	120	< 7 mm
22AA	44	6	80	< 7 mm
22AB	44	6	80	< 7 mm

23AA	73	6	40	> 7 mm
23AB	73	6	40	> 7 mm
24AA	44	6	80	Full Mixed
24AB	44	6	80	Full Mixed
